# C-reactive protein as robust laboratory value associated with prognosis in patients with stage III non-small cell lung cancer (NSCLC) treated with definitive radiochemotherapy

**DOI:** 10.1038/s41598-024-64302-2

**Published:** 2024-06-14

**Authors:** Cedric Richlitzki, Marcel Wiesweg, Martin Metzenmacher, Nika Guberina, Christoph Pöttgen, Hubertus Hautzel, Wilfried E. E. Eberhardt, Kaid Darwiche, Dirk Theegarten, Clemens Aigner, Servet Bölükbas, Martin Schuler, Martin Stuschke, Maja Guberina

**Affiliations:** 1 Department of Radiation Oncology, University Hospital, LMU Munich, Munich, Germany; 2 Department of Radiotherapy, West German Cancer Center, University Hospital Essen, Essen, Germany; 3 National Center for Tumor Diseases (NCT) West, Essen, Germany; 4 Department of Medical Oncology, West German Cancer Center, University Hospital Essen, Essen, Germany; 5 Division of Thoracic Oncology, University Medicine Essen – Ruhrlandklinik, Essen, Germany; 6 Department of Nuclear Medicine, West German Cancer Center, University Hospital Essen, University Duisburg-Essen, Essen, Germany; 7 German Cancer Consortium (DKTK), Partner Site University Hospital Essen, Essen, Germany; 8 Department of Pulmonary Medicine, Section of Interventional Pneumology, West German Lung Transplantation Center, University Medicine Essen – Ruhrlandklinik, Essen, Germany; 9 Institute of Pathology, University Hospital Essen, Essen, Germany; 10 Department of Thoracic Surgery, Comprehensive Cancer Center, Medical University of Vienna, Vienna, Austria; 11 Department of Thoracic Surgery, Medical Faculty, West German Cancer Center, University Hospital Essen, Ruhrlandklinik, Tueschner Weg 40, 45239 Essen, Germany; 12 Bavarian Cancer Research Center (BZKF), Munich, Germany

**Keywords:** C-reactive protein (CRP), Stage III non-small cell lung cancer (NSCLC), Definitive radiochemotherapy, Laboratory values, Overall survival, ESPATUE trial, Cancer, Non-small-cell lung cancer, Biomarkers, Diagnostic markers, Predictive markers, Prognostic markers, Medical research, Outcomes research

## Abstract

To evaluate the prognostic value of biomarkers from peripheral blood obtained as routine laboratory assessment for overall survival in a cohort of stage III non-small cell lung cancer (NSCLC) patients treated with definitive radiochemotherapy at a high-volume cancer center. Seven blood biomarkers from 160 patients treated with definitive radiochemotherapy for stage III NSCLC were analyzed throughout the course treatment. Parameters were preselected using univariable and multivariable proportional hazards analysis and were assessed for internal validity using leave-one-out cross validation. Cross validated classifiers including biomarkers in addition to important clinical parameters were compared with classifiers containing the clinical parameters alone. An increased C-reactive protein (CRP) value in the final week of radiotherapy was found as a prognostic factor for overall survival, both as a continuous (HR 1.099 (1.038–1.164), *p* < 0.0012) as well as categorical variable splitting data at the median value of 1.2 mg/dl (HR 2.214 (1.388–3.531), *p* < 0.0008). In the multivariable analysis, the CRP value-maintained significance with an HR of 1.105 (1.040–1.173) and *p*-value of 0.0012. The cross validated classifier using CRP at the end of radiotherapy in addition to clinical parameters separated equally sized high and low risk groups more distinctly than a classifier containing the clinical parameters alone (HR = 2.786 (95% CI 1.686–4.605) vs. HR = 2.287 (95% CI 1.407–3.718)). Thus, the CRP value at the end of radiation therapy has successfully passed the crucial cross-validation test. The presented data on CRP levels suggests that inflammatory markers may become increasingly important during definitive radiochemotherapy, particularly with the growing utilization of immunotherapy as a consolidation therapy for stage III NSCLC.

## Introduction

Lung cancer is the leading cause of cancer-related death worldwide^1^. Stage III NSCLC is highly heterogeneous, encompassing a wide spectrum of disease distribution. At the time of initial diagnosis, approximately 25% of patients with NSCLC are in stage III, for which definitive radiochemotherapy is a standard treatment option. Historically, median survival after radiochemotherapy has ranged from 15 to 30 months^2^. Long term survival and progression-free survival of definitive radiochemotherapy could be improved by consolidation immune check point inhibitors^3,4^. Despite the advent of check-point inhibitors, overall survival of stage III patients treated with definitive radiochemotherapy remains limited according to real-world data on progression-free survival, which was 48.2% in the PACIFIC-trial^5^. In patients with stage III EGFR-mutant NSCLC the combination with newer-generation tyrosine kinase inhibitors and radiochemotherapy are promising but recurrences can occur predominantly in the brain^6^. Likewise immunotherapy has determined unprecedented long-term responses in NSCLC^7^ and concomitant therapies can highly influence its impact^8^.

Heterogeneity in outcome requires prognostic parameters. Determining the optimum first-line therapy for advanced non-small cell lung cancer, e.g. with high PD-L1 expression, is currently being analyzed in larger studies. The precise determination of valuable biomarkers is essential and a prerequisite to further optimize treatment^9–12^.

Laboratory parameters, such as leukocytosis^13^, lymphocytopenia^14^, neutropenia^15^, elevation of lactate dehydrogenase (LDH)^16^, C-reactive protein (CRP) changes^17–19^, thrombocytosis^20^, and low hemoglobin (Hb)^21^, have been identified as prognostic factors in stage III NSCLC patients. In a prior investigation we found in de-novo oligo-metastatic disease that severe comorbidity, ECOG performance status, sex and pre-treatment serum CRP level as the most important factors in the univariable analysis in consecutive cohort treated at our centre^22^.

This study retrospectively analyzed data from all consecutive patients (n = 160) who underwent definitive radiochemotherapy for unresectable stage IIIA-C NSCLC at the Department of Radiation Oncology at a high-volume lung cancer center, between January 2017 and February 2020. The objective of the research is to examine laboratory parameters during treatment and draw inferences to predict or forecast overall survival.

### Endpoints

The study's primary endpoint was to determine the meaning of routinely measured laboratory values on overall survival (OS). The secondary objective was to assess the frequency of abnormal laboratory results during treatment.

## Materials and methods

All patients with confirmed locally advanced NSCLC who started definitive radiochemotherapy at a high-volume cancer center between 01/2017 and 02/2020 were included according to a consecutive study design^4^. The local ethics committee of the Faculty of Medicine approved the data collection and analysis for this retrospective study (study identifier: 21–10,033-BO and 21–10,378-BO). The research was conducted in accordance with the 1964 Declaration of Helsinki^4^. Informed consent was waived due to the retrospective data analysis with anonymized data.

The analyses were carried out with the Attelica CH system Immunoturbidimetry (Atellica® Solution – Siemens Healthineers Deutschland) (last accessed on 13.02.2024)^23^. The Atellica CH C-Reactive Protein_2 (CRP_2) latex reagent is a suspension of polystyrene latex particles coated with anti-CRP antibodies^24^. By the mixture of serum containing CRP with the latex reagent, agglutination occurs, resulting in an increase in turbidity. This turbidity was measured at 571 nm. The CRP concentration in serum was determined using a calibration curve generated with the calibrators^24^. The values were collected within 4 h and therefore a high stability was granted^24^.

### Confirmation of diagnosis and TNM-staging

Definitive histopathologic diagnosis of NSCLC was made by an accredited and experienced pathology center. TNM classification was performed according to the eighth edition of the Union for International Cancer Control/American Joint Committee on Cancer classification^25^. Tumor staging and exclusion of distant metastases were performed by computed tomography (CT) and^18^F-FDG PET/CT prior to treatment initiation. In addition, mediastinal lymph node staging was performed by systematic endobronchial ultrasound-guided transbronchial needle aspiration. Clinical performance was evaluated with dedicated pulmonary function testing (spirometry, lung diffusing capacity for carbon monoxide, ventilation-perfusion scan) and cardiac assessment (stress ECG and echocardiography). All patients were scheduled to receive a CT or MRI scan of the brain. All patients deemed potentially resectable at a pretreatment conference prior to induction chemotherapy were discussed in a second interdisciplinary tumor board during the last week prior to a cumulative radiation dose of nearly 46 Gy based on available interim CT or^18^F-FDG PET/CT and clinical performance. If the tumor was ultimately deemed technically, functionally, or prognostically unresectable (higher risk of R1 or R2 resection), patients received definitive radiochemotherapy^4^.

### Treatment planning and strategy

In a curative intention-to-treat setting, patients without serious contraindications received approximately two to four cycles of cisplatin and paclitaxel/vinorelbine or etoposide-based induction chemotherapy^4^. After re-evaluation by a tumor board, patients deemed unresectable underwent definitive concurrent radiochemotherapy, considering local tumor extent and overall performance status^4^. Depending on general condition and laboratory findings, two cycles of concurrent cisplatin and vinorelbine based chemotherapy with 50 mg/m^2^ cisplatin and 20 mg/m^2^ vinorelbine were prescribed and administered on days 2 and 9, with 40 mg/m^2^ cisplatin and 15 mg/m^2^ vinorelbine administered on days 29 and 36. Patients with renal impairment and those intolerant of concurrent doublet chemotherapy received either carboplatin and vinorelbine or cisplatin or carboplatin monotherapy. Three patients with large cell neuroendocrine tumors received a modified chemotherapy protocol, and because induction chemotherapy began with cisplatin and etoposide, these patients continued with the same medications. Radiotherapy was administered with either a fractionated dose of 1.5 Gy/F twice daily, with a minimum of 6 h between fractions, to a total dose of 45 Gy, followed by conventional fractionation to 61–65 Gy analog the ESPATUE Trial^26–28^, or a fractionated dose of 2 Gy/F once daily, 5 days a week^4^.

Treatment was delivered using either volumetric modulated arc therapy or static field intensity modulated radiotherapy with daily image-guided radiotherapy on a 6-degree-of-freedom table and a TrueBeam linear accelerator (Varian, CA, USA). Pretreatment audiovisual respiratory training and a respiratory gating system allowed daily online imaging and reproducible, precise patient positioning from fraction to fraction. Treatment plans were generated with a planning CT after delineation of gross tumor volume, clinical target volume, and planning target volume using the Eclipse treatment planning system and the Acuros XB algorithm (Varian). Margins were selected based on reproducibility, considering individual respiratory phases. Organs at risk constraints were a maximum dose of 42 Gy to the spinal cord and an average dose of 19 Gy to the lungs. The dose covering 98% of the planned target volume had to be greater than 95% of the total prescribed dose.

Patients with PD-L1-expressing tumors have been offered durvalumab consolidation after completion of radiochemotherapy by an early access program or since the approval of durvalumab, according to the eligibility criteria of the PACIFIC trial. Durvalumab was routinely administered at 10 mg/kg intravenously every 2 weeks for up to 12 months (24 cycles). Alternative monthly dosing regimens were also offered. Reasons for early discontinuation included acute toxicity, adverse events, tumor progression and tumor relapse. In the event of distant or local tumor recurrence, systemic therapy was offered according to the standard of care as defined by national and international guidelines (e.g., PD-L1 antibody alone or in combination with chemotherapy) whenever possible and medically appropriate.

### Statistical analysis

SPSS Statistics Version: 28.0.0.0 (190) (IBM, NY, USA) and SAS Version: 14.1 (SAS Institute, NC, USA) were used for descriptive statistics and statistical analysis. Overall survival was defined from the time of radiotherapy start to death. Survival time analyses were performed using the Kaplan–Meier method. Kaplan–Meier curves were compared with the log-rank test. In addition, the influence of quantitative or categorical factors on overall survival was examined using proportional hazards (ph) analysis and the corresponding hazard ratio (HR) with its 95% confidence intervals (CI) was reported. A *p*-value < 0.05 was considered as of importance. Prognostic laboratory parameters that showed a *p*-value < 0.05 in the univariable analysis were analyzed simultaneously in the multivariable analysis together with important clinical cofactors. The proportional hazards assumption using the Kolmogorov-Type Supremum Test and no significant deviations were detected at *p* < 0.05, if not reported otherwise. The deviations of the functional form of a linear quantitative covariate, were also examined with a supremum test.

The cofactors found to be important in the multivariable analysis were further analyzed using leave-one-out cross validation. This technique is intended to prevent overoptimistic predictions^29^. Here, we used with the LOOCV SAS Macro described by Rushing et al.^30^. This Macro is available for download at https://sites.google.com/view/herbpang/sassurvloocv (last accessed on 28.10.2023)^30^. In each leave-one-out loop, the best four- or five-parameter prognostic model with the highest Chi2-(X^2^) statistic in the score test was determined on the training dataset with n-1 patients using proportional hazard analysis^30,31^. In this study, the classifier contained a set of important clinical factors and in addition one laboratory parameter. Since the model with laboratory data is an extension of the clinical model and thus the models are branched and the difference in the log-rank statistics for the separation of the high- and low-risk groups in both models has importance for the model comparison^29^.

### Ethical approval

The local ethics committee of the Faculty of Medicine approved the data collection and analysis for this retrospective study (study identifier: 21–10,033-BO and 21–10,378-BO). The research was conducted in accordance with the 1964 Declaration of Helsinki. Informed consent was waived due to the retrospective data analysis with anonymized data.

## Results

### Patient cohort and eligibility

**Figure 1:**
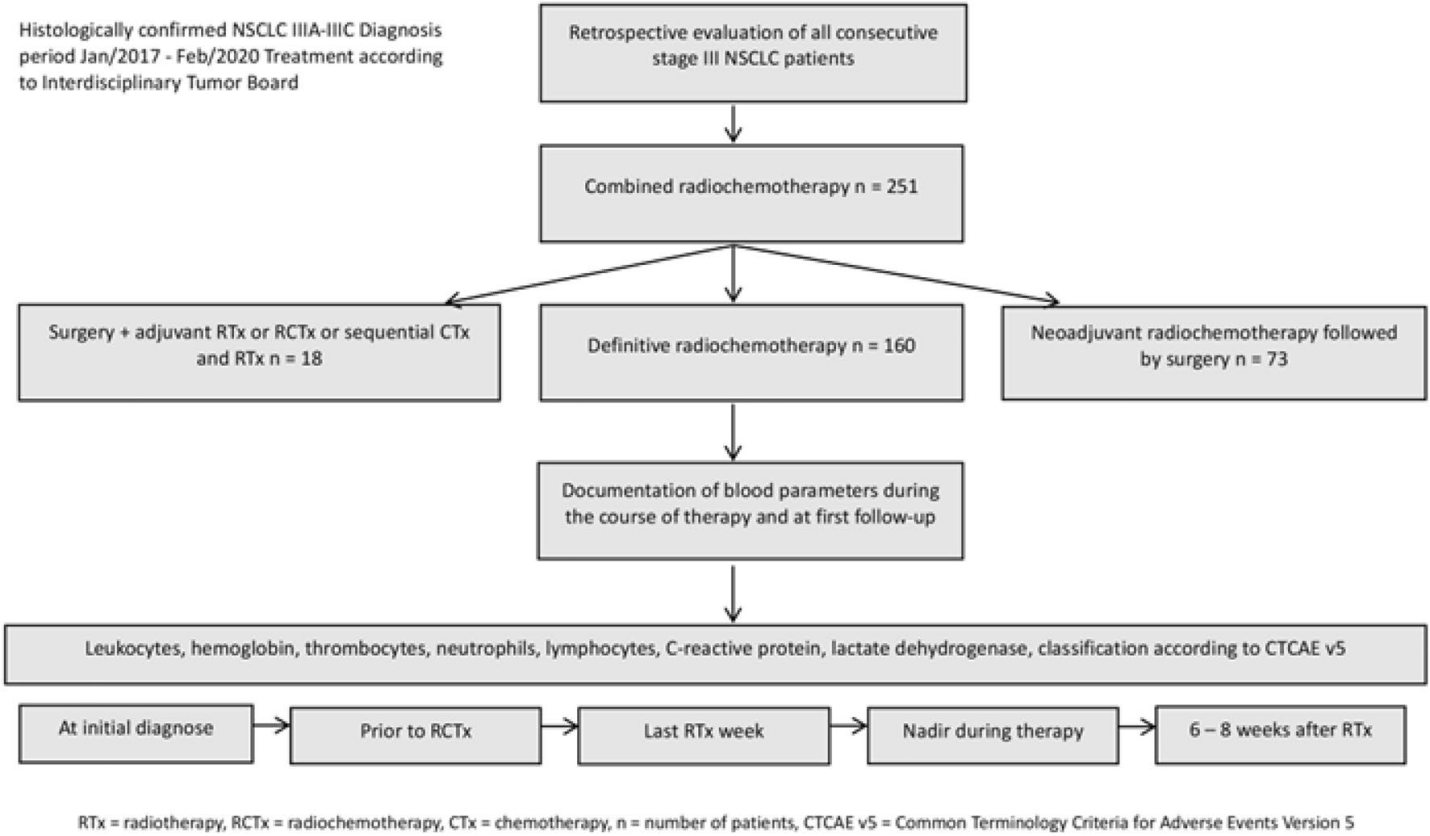
Flow chart study design (definitive radiochemotherapy and documentation of laboratory parameters).

From January 2017 to February 2020, a total of 160 patients (51 women and 109 men; median age: 66 years; range: 44–86) with unresectable locally advanced stage III NSCLC received definitive radiochemotherapy at the Department of Radiation Oncology (Fig. 1 flow chart). All patient cases were discussed in the interdisciplinary tumor board before the start of induction chemotherapy and additionally during the last week of radiotherapy before the neoadjuvant radiation dose of 46 Gy was reached. Twenty-two and seven patients had an ECOG performance status of grade 2 or 3, respectively. Patient characteristics are listed in Table 1.

**Table 1:** Patient characteristics.

Descriptive characteristics NSCLC stad. IIIA-IIIC Definitive radiochemotherapy		
Patient characteristics	N = 160	%
Age (years) Median	66,5 (43,6–86,4)	
Sex		
Male	109	68,2
Female	51	31,9
**T**NM-Stage		
cT 1–2	38	23,8
cT 3	29	18,1
cT 4	93	58,1
T**N**M-Stage		
cN 0–1	44	27,5
cN 2	76	47,5
cN 3	40	25
UICC-Stage		
IIIA	69	43,1
IIIB	63	39,4
IIIC	28	17,5
Grading		
G1	2	1,9
G2	30	29,1
G3	71	69,0
Histology		
Squamous cell carcinoma	79	49,4
Adenocarcinoma	59	36,9
Other	22	13,7
RT-Dose (Gy) Median	63,7 (53,5–72)	
≤ 54 Gy	1	0,6
> 54—≤ 66 Gy	150	93,8
> 66—< 72 Gy	9	5,6
Durvalumab		
Yes	39	24,4
No	121	75,6
ECOG Performance Status		
0	38	23,75
1	93	58,12
2	22	13,75
3	7	4,38
Death		
Yes	84	52,5
No	72	47,5

RT = Radiotherapy, UICC = Union for international cancer control, Gy = Gray, TNM = Tumor (T), Nodes (N), Metastases (M), N/A = Not available OS = Overall survival, ECOG = Eastern cooperative oncology group.

Median follow-up was 31.1 months. A total of 91% of patients who were treated with definitive radiochemotherapy received induction chemotherapy. A major reason for not using induction chemotherapy were poor performance status, comorbidities or symptoms as bleeding leading to the preference of immediate concurrent radiochemotherapy. All patients received a total radiation dose greater than 53.5 Gy, and 94% of patients received a dose of 60 Gy or greater (mean dose: 63.7 Gy; range: 53.5–72.0). A total of 83% of patients received concurrent cycles of platinum-based chemotherapy. A total of 14% of patients who were deemed intolerant to concurrent doublet chemotherapy received either cisplatin or carboplatin monotherapy according to their renal function, and 3% were treated with definitive radiotherapy alone (after induction chemotherapy) due to comorbidities. After definitive radio chemotherapy a total of 39 patients received durvalumab consolidation either as early access or after drug approval following publication of the PACIFIC trial (NCT02125461)^32^. Overall of the whole group of 160 consecutive patients at 36 months of follow-up was 42.6% ± 0.45%.

Seven blood parameters were documented at four predetermined intervals throughout treatment and follow-up. Additionally, nadir values were recorded (Fig. 1 flow chart). These parameters were classified according to Common Terminology Criteria for Adverse Events (CTCAE) Version 5 standards^33^. The corresponding grades are summarized in tables located in the appendix. No grade 5 adverse events were reported. For analysis, adverse events were grouped into three grades (Grade 1–2, Grade 3, and Grade 4).

Most patients (71.3%) displayed a grade 1–2 CRP elevation at initial diagnosis, followed by 44.4% with grade 1–2 anemia and 35.8% with a grade 1–2 LDH elevation. Incidences of grade 3 and 4 laboratory changes were uncommon. At the time of initial diagnosis, the median of all laboratory parameters, apart from CRP (1.5 mg/dl), fell within the physiological range (Tables 4, 5, 6, 7, 8 appendix).

After induction chemotherapy, before the start of radiochemotherapy the most common grade 1–2 adverse event observed was anemia in 79.6% of patients, followed by an increase in CRP grade 1–2 in 55.3% of patients and increased LDH grade 1–2 in 43.6% of patients. There was a 76% increase in patients with grade 1–2 anemia, while the prevalence of grade 1–2 CRP decreased by 26.32%. Following induction chemotherapy, most blood parameters had median values within the physiological range. The median hemoglobin value for both males and females (11.9 g/dl) and the median CRP value (0.65 mg/dl) were exceptions and outside the normal range (Tables 4, 5, 6, 7, 8 appendix).

During the final week of radiotherapy, Lymphocytopenia was identified as the most prevalent grade 3 and grade 4 adverse event, affecting 39.5% and 7% of patients respectively. Anemia registered as the most common grade 1–2 adverse event, affecting 87% of patients, followed by elevated CRP in 65.7% and lymphopenia in 44.2% of patients. Most blood parameters remained within physiological levels, as compared to the median. However, the hemoglobin level (10.75 g/dL), CRP (1.2 mg/dL), and, for the first time, lymphocyte count (520/µL) were all outside of the physiological range. The nadir values for five blood parameters, namely leukocytes, hemoglobin, platelets, neutrophils, and lymphocytes, were recorded throughout the course of therapy (Tables 4, 5, 6, 7, 8 appendix).

As part of the initial follow-up exam six to eight weeks after therapy completion, additional documentation of blood parameters was conducted. The prevailing grade 1–2 side effect was anemia in 76.7% of the patients, while 61.8% exhibited elevated CRP, 54.2% developed lymphocytopenia, and 48.1% displayed pathologically elevated LDH. Leukopenia and elevated CRP were the most observed grade 3 adverse effects, with 15.6% and 10.1%, respectively. The median values of lymphocytes (820/µl) and CRP (1.9 mg/dl) fall within the pathological range.

Significant association with overall survival was found for leukocyte counts and CRP values in univariable selection proportional hazards analysis at alpha = 0.05.

**Figure 2:**
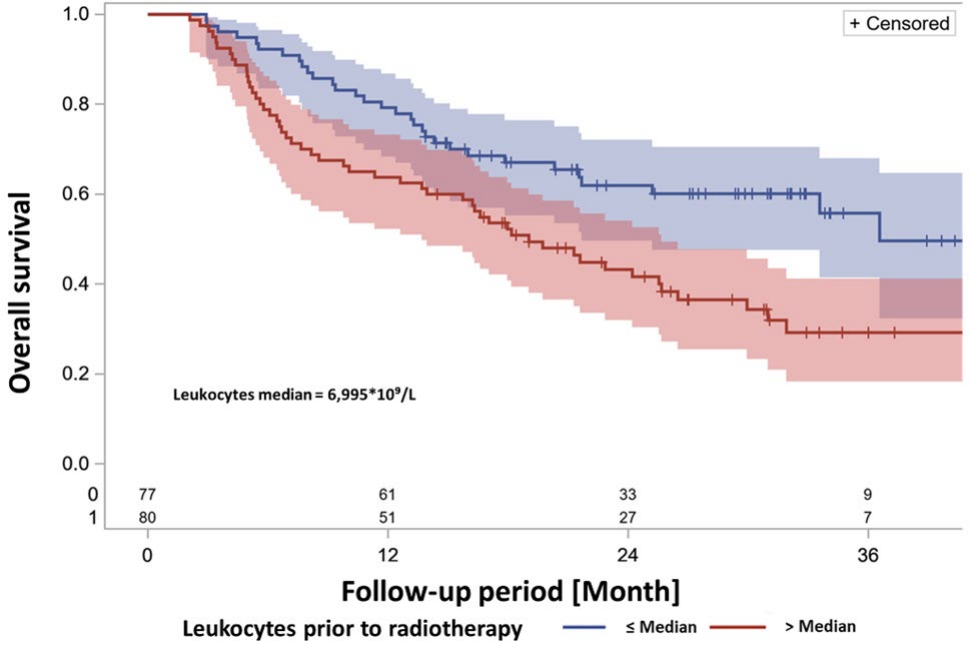
Kaplan–Meier survival curve, leukocyte count prior to radiotherapy dichotomized according to the median. There is a statistically significant difference between the two groups in terms of survival (log-rank test *p* = 0.0030, hazard ratio HR = 1.940 (95% CI 1.243—3.030), *p* = 0.0035, N = 157 patients with this observed parameter.

Regarding the leukocyte count, the univariable ph regression analysis yielded the strongest finding of a prognostic impact of the leukocyte count prior to the start of radiotherapy. Univariable analysis results are depicted in Table 2. Figure 2 exhibits survival curves for patients with leukocyte counts either above or equal to or below the median value.

**Table 2:** Univariable analysis, leukocyte count during therapy.

Univariable analysis cox regression: influence of leukocytosis on OS		
(1) Continuous, quantitative value		
Measurement time: (two groups)	HR (95% CI)	*p*-Wert
First diagnose	1.027 (0.962–1.096)	0,4322
Prior to RCTx start	1,082 (1,023–1.145)	0,0059
Leukocyte nadir	1.066 (0.940–1.209)	0.0657
Last RCTx week	1.095 (0.989–1.212)	0.0814
6–8 weeks after RCTx	1.200 (1.073–1.314)	0.0013

(2) Cut-off: median		
Measurement time: (two groups)	HR (95% CI)	*p*-Wert
First diagnose	1,258 (0.817–1.937)	0,2978
Prior to RCTx start	1,940 (1.243–3.030)	0,0035
Leukocyte nadir	1.506 (0.974–2.330)	0.0557
Last RCTx week	1.679 (1.057–2.667)	0,0281
6–8 weeks after RCTx	1.889 (0.946 -3,774)	0,0715

HR = Hazard ratio, CI = Confidence interval, OS = Overall survival *10.2* × *109/L,* supremum test on proportional hazard analysis.

**Figure 3:**
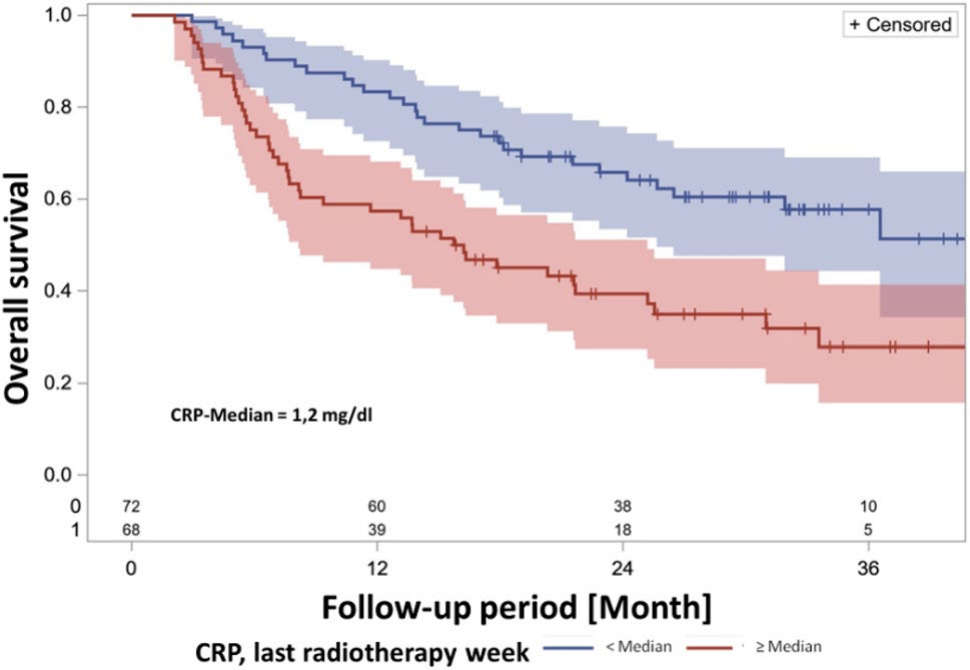
Kaplan–Meier survival curve, CRP in the last RTx week, dichotomized by median. There is a statistically significant difference between the two groups in terms of survival (log-rank test *p* = 0.0012, hazard ratio HR = 2.214 (95% CI 1.388—3.531) *p* = 0.0008, N = 140 patients with this observed parameter).

**Figure 4:**
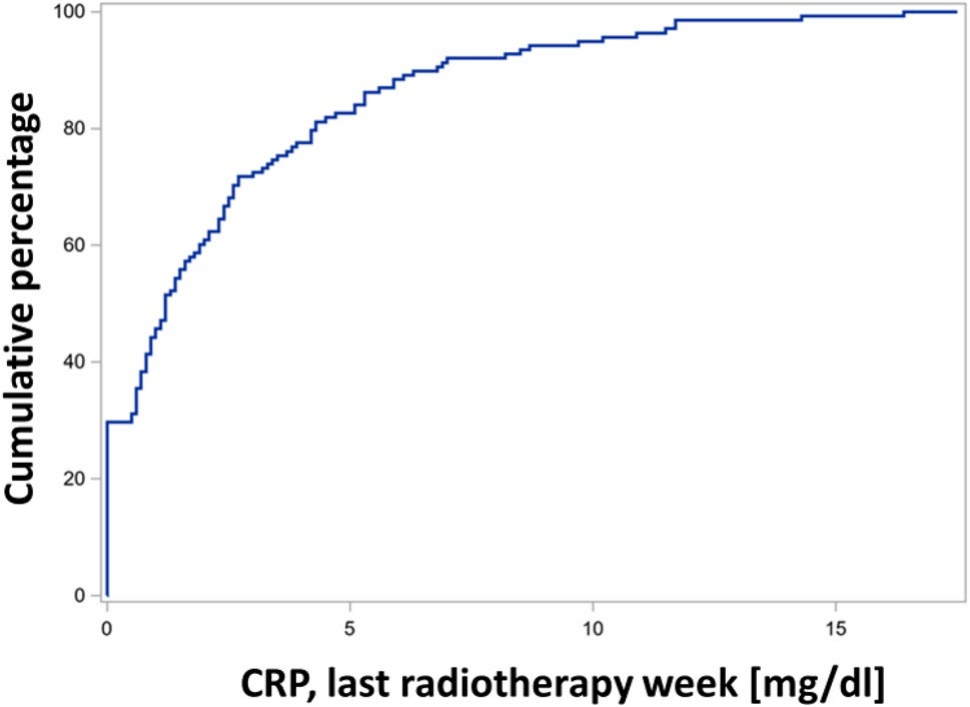
Empirical distribution function of CRP values in the serum of patients in the last week of radiotherapy, CRP unit: mg/dl.

In the ph univariable analysis with CRP value as a continuous variable, the last week's CRP value during radiotherapy was identified as a prognostic factor for overall survival. The CRP value at the end of radiotherapy dichotomized at the median also highlighted the association between higher CRP values and decreased survival (Table 3, Fig. 3). Figure 4 displays the empirical distribution function of CRP levels in the serum of patients during their final week of radiotherapy. This demonstrates the proportion of patients with CRP levels less than or equal to the value on the corresponding x-axis in the final week of radiotherapy. The median CRP level was 1.2 mg/dl.

**Table 3:** Univariable analysis, CRP during therapy.

Univariable analysis: influence of CRP on the OS		
Continuous, quantitative value		
Measurement time:	HR (95% CI)	*p*-value (sig. < ,05)
First diagnose	1,049 (1.010—1.091)	0,0144
Prior to RCTx start	1,007 (0.949—1.067	0,8265
Last RCTx week	1,099 (1,038—1,164)	0,0012
6–8 weeks after RCTx	1,048 (0.996—1.103)	0.0732

Cut-off median		
Measurement time:	HR (95% CI)	*p*-value (sig. < ,05)
First diagnose	1,273 (0,829—1,954)	0.2705
Last RCTx week	2,214 (1,388—3,531)	0,0008

HR = Hazard ratio, CI = Confidence interval, OS = Overall survival, RTx = Radiotherapy, CRP = C-reactive protein.

The next step was to analyze each candidate’s blood markers, passed the univariable analysis in multivariable analysis together with import clinical factors known to affect prognosis, i.e. ECOG status 1 vs. 0, ECOG status 2 vs 0, age, Durvalumab consolidation (Yes or no). The clinical factors were previously identified in our previous work on this^4^. Table 4 shows the results of multivariable analysis and both markers, CRP during last week of radiotherapy and Leukocyte count prior to radiotherapy showed *p* values < 0.05.

**Table 4:** Results of the multivariable proportional hazards analysis.

Parameter	*p*-Value	HR (95% CI)
Durvalumab Yes/No	0,0034	0,281 (0,120–0,656)
ECOG 1 vs. 0	0,1922	1,625 (0,783–3,372)
ECOG 2 vs. 0	0,0052	3,194 (1,413–7,217)
Age	1,1847	1,019 (0,991–1,048)
CRP last RT-Week	0,0012	1,105 (1,040–1,173)
Leukocytes before RT-week	0,0204	1,766 (1,092–2,856)

RT = Radiotherapy, CRP = c-Reaktive protein, ECOG = Eastern cooperative oncology group.

**Figure 5:**
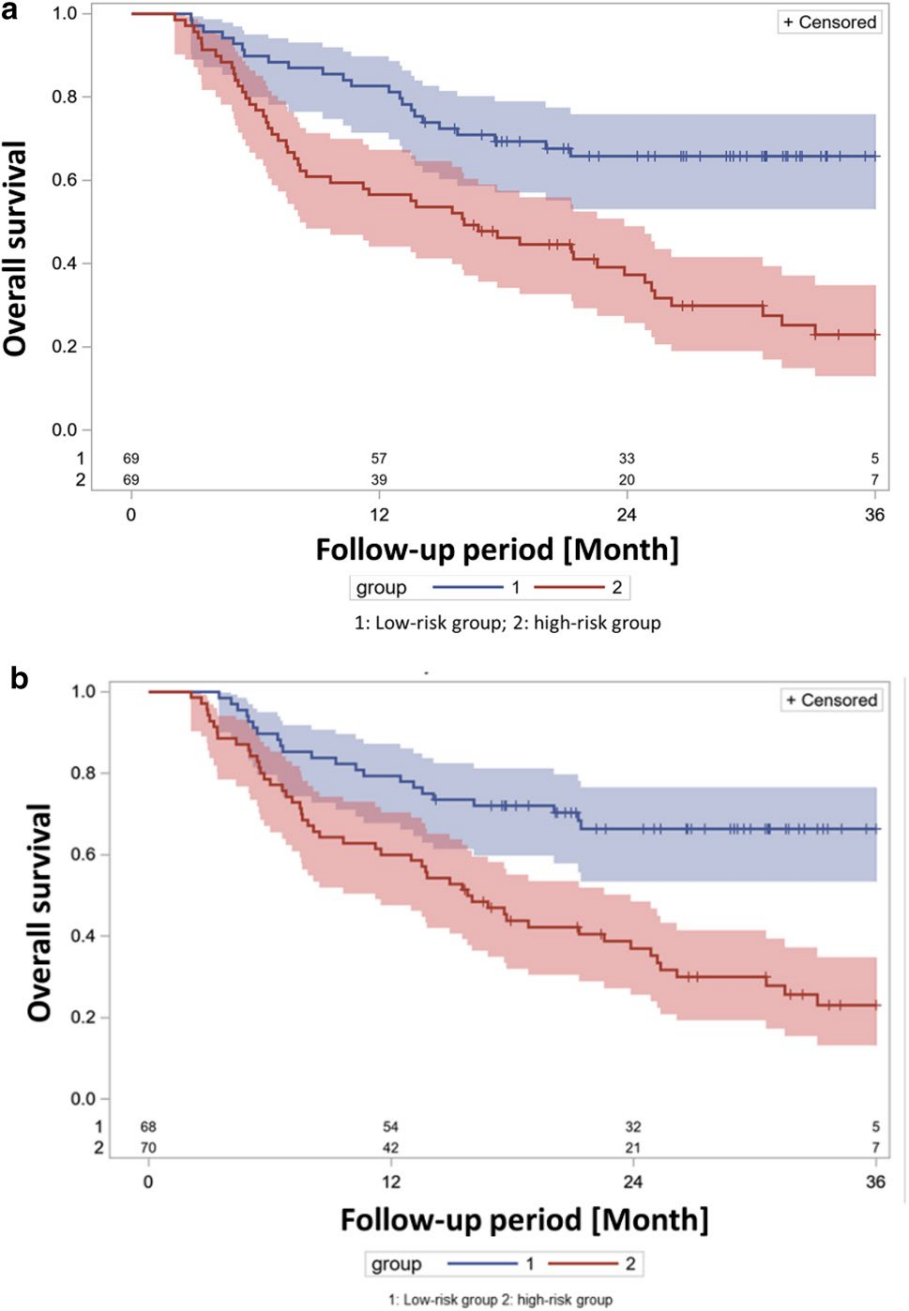
(**a**) Leave-one-out cross-validated Kaplan–Meier survival curves for the high (red) and low (blue) risk groups according to the best 4-parameter classifier with only clinical factors; durvalumab application, ECOG 2 versus 0, ECOG 1 versus 0 and age. In the log-rank test, the X^2^ value for a difference is X^2^ = 11.7853 (*p* = 0.0006). (**b**) Leave-one-out cross-validated Kaplan–Meier survival curves for the high (red) and low (blue) risk groups according to the 5-parameter classifier from the 4 clinical factors; durvalumab application, ECOG 2 versus 0, ECOG 1vs 0 and age as well as the laboratory parameter CRP in the last RT week In the log-rank test, the X^2^ value for a difference is X^2^ = 17.4117 (*p* < 0.0001).

In a next step of parameter selection, leave-one-out cross validation was used to assess the internal validity of the biomarkers that passed multivariable analysis. Cross-validated survival curves were generated for the high-risk and low-risk groups using a 4-parameter classifier based on four clinical factors: age, ECOG (status 1 vs. status 0 prior to therapy), ECOG (status 2 vs. status 0 prior to therapy), and durvalumab consolidation (yes/no). During each leave-one-out-loop, a patient was classified as high-risk or low-risk based on the median of the best 4-parameter classifier in the training dataset, with one patient excluded each time. The log-rank test revealed a significant difference in survival curves (X^2^ = 11.7853, *p* = 0.0006), the hazard ratio between the high and low risk groups in the proportional hazards analysis was calculated to be HR = 2.287 (95% CI 1.407—3.718) (Fig. 5a).

Then, the effect of laboratory parameters additional to the 4 clinical parameters was analyzed using leave-one-out cross validation. Five parameter classifiers were constructed adding either CRP at the end of radiotherapy or Lymphocyte count prior to the start of radiotherapy to the 4 clinical parameters. CRP alongside with age, ECOG status prior to therapy (1 vs. 0 and 2 vs. 0), and durvalumab consolidation (yes/no) resulted in a cross-validated classifier, separating the high-risk and low-risk groups markedly (log rank test X^2^ = 17.4117, *p* =  < 0.0001, Fig. 5b). The hazard ratio between the high-risk and low-risk groups was HR = 2.786 (95% CI: 1.686—4.605). Comparing the X^2^ values of the log-rank statistics of the two nested models with the CRP values and without blood parameters^16^, the resulting X^2^ was 5.6264 (*p* = 0.0177, 1 degree of freedom). Therefore, the CRP value obtained at the end of radiotherapy successfully passed the critical cross-validation test, and it is recommended for evaluation in future studies.

In addition, the 5-parameter leave-one-out classifier including leukocyte count prior to radiotherapy was studied. The log-rank test statistic for the difference in survival curves between high- and low-risk groups was (X^2^ = 15.5778, *p* < 0.0001) and the hazard ratio between the high- and low-risk groups in the proportional hazards analysis was 2.592 (95% CI 1.586—4.234). The improvement in comparison to the classifier with clinical parameters alone was not large enough, to announce leukocyte prior to radiotherapy as a prognostic parameter that has passed cross validation.

## Discussion

CRP, as an acute phase protein, is an established marker for inflammatory processes^34^. An elevation in serum CRP is observed in malignant tumors and is associated with several mechanisms leading to worse prognosis of tumor patients^35^. Elevated CRP is a hallmark of cachexia and this in turn is a mechanism associated with worse prognosis in lung cancer and other tumors^18,36–39^. Additionally, it has been demonstrated to impact tissue invasion and metastasis^40^. CRP, a widely available biomarker, has been studied in other protocols for its ability to provide information about tumor prognosis. In a pan-cancer analysis, it was announced as a negative prognostic marker for overall survival in advanced cancer patients^41^. The findings of more recent studies on patients with predominately stage IV NSCLC also indicated an association between higher CRP levels and shorter survival time in patients with distant metastases treated with chemotherapy, immunotherapy or both^42–47^.

In a prior investigation we found that severe comorbidity, ECOG performance status, sex and pre-treatment serum CRP level as the most important factors in the univariable analysis in a consecutive cohort of de-novo oligometastatic NSCLC treated at our centre^22^. Also other study groups found the predictive value of baseline CRP levels on the efficacy of chemotherapy plus immune checkpoint inhibitors in patients with advanced lung squamous cell carcinoma. Here, a high CRP level was identified as an independent risk factor for poor PFS through multivariate-adjusted analysis for the efficacy of combination therapy with chemo-immunotherapy not for chemotherapy alone^48^.

Nevertheless, none of our patients received chemotherapy as a monotherapy, but combined with radiotherapy with or without immune check point inhibition. There is some evidence that inflammation is connected to an immunosuppressive tumor micro milieu and is associated with up-regulation of the immunosuppressive adenosine pathway^49^. In patients with advanced NSCLC with high PD-L1 expression without targetable driver mutations, both first line chemo-immunotherapy and immune check-point monotherapy as first-line treatment for advanced NSCLC result in similar overall survival according recent meta-analyses of randomized trials. However randomized head to head analyses still are lacking^9^. Biomarker-guided targeted therapies may offer valuable advantages for patients as they have proven to be of great benefit in metastatic tumors^50–52^. Additional prognostic factors for treatment recommendation gain great importance for all subgroups of patients^50,53,54^.

Furthermore, CRP is also associated with the risk of side effects of immunotherapy as pneumonitis or hepatotoxicty^55^. Data on the prognostic value of CRP for stage III NSCLC patients treated with concurrent radiochemotherapy with or without durvalumab consolidation are rather limited. For a rather homogeneous group of 89 stage III NSCLC treated with concurrent or sequential radiochemotherapy between 2006 and 2013, Mitsuyoshi et al. found a larger CRP level ≥ 0.3 mg/dl to be associated with shorter OS^56^. Tolia et al. found a predictive value of CRP for a heterogeneous cohort of stage I-IV NSCLC patients treated with definitive radiochemotherapy^17^. Guckenberger et al. analyzed longitudinal CRP levels up to 12 months in stage III NSCLC patients treated with concurrent radiochemotherapy and durvalumab consolidation in a smaller cohort of 22 patients but did not find an increase of the percentage of patients with CRP elevation > 5 mg/dl during the course of therapy and follow-up. At this cut-point the percentage of patients with elevated CRP stayed below 10%^57^. In the present study, more than 20% of patients had a CRP level > 5 mg/dl during the last week of radiotherapy. The present study included a more recent and larger group of consecutive stage III NSCLC treated with definitive radiochemotherapy. Induction chemotherapy was a preferred option and was used in 91% of patients. Durvalumab consolidation was offered after durvalumab approval to all patients with a PD-L1 tumor proportion score ≥ 1% or by an early access program. CRP value at the end of radiotherapy was a strong prognostic factor using univariable and multivariable analysis. Furthermore, it successfully passed cross-validation for internal validity and is announced for assessment in subsequent research. That CRP measurements during therapy can reveal additional information above the baseline value has been also shown by some other studies on immunotherapy in advanced NSCLC^58–61^.

The study's strength lies in the significant sample size of patients with strictly defined inclusion criteria, similar tumor spread, and uniform treatment protocols treated at a high-volume lung tumor center. However, limitations exist due to the study's retrospective design. In addition external validation by an independent larger cohort was not conducted in stage III but up to now CRP at base line was also analyzed so far in oligometastatic NSCLC patients treated with thoracic radiochemotherapy and found to be prognostic^22^. Furthermore, various other biomarkers and prognostic parameters should be investigated in more detail as e.g. several study groups tried to define connected imaging, prognostic and predictive biomarkers for response and monitoring after neoadjuvant therapy^62,63^ or stereotactic radiotherapy and immunotherapy in locally advanced or metastatic lung cancer^64^. Zafra et al. found increased presence of CD8 + PD1 + cells with SABR in patients with adequate tumor response^64^. In addition, study results found the association and interaction between decreased albumin levels, liver function and immunotherapy effect^47,65^. To date, elevated blood parameters have not significantly influenced clinical treatment decisions for radiochemotherapy^27,66–69^. Among the various blood parameters analyzed, the CRP value at the end of radiotherapy was determined to be a promising biomarker. Overall, patients with locally advanced lung cancer can be separated in equally sized good and bad prognosis with large differences in prognosis. Beneath clinical variables, as Eastern Cooperative Oncology Group (ECOG) performance status, age, and durvalumab consolidation inflammation markers can play an important role. The presented data show, that longitudinal repeated measures of inflammation markers can carry more information than base line measures alone. The importance of biomarkers for stage III NSCLC treated with definitive radiochemotherapy might even increase in the area of additional immunotherapy.

### Supplementary Information

Supplementary Information.

## Data Availability

The data sets generated and analyzed in this study are included in this published article. Additional data are available upon request to the corresponding author.

## Abbreviations

CRP: C reactive protein
LOOCV: Leave-on-out cross validation
NSCLC: Non-small cell lung cancer
CT: Computer tomography
MRI: Magnetic resonance imaging
^18^F-FDG PET/CT: Fludeoxyglucose ^18^F (FDG) positron-emission computer tomography
Gy: Gray
CI: Confidence interval
HR: Hazard ratio
CTCAE: Common terminology criteria for adverse events
LDH: Lactate dehydrogenase
ECOG: Eastern cooperative oncology group

## Code availability

The SAS Survival LOOCV Macro is available for download: https://sites.google.com/view/herbpang/sassurvloocv, last accessed on 28.10.2023^30^.

## References

[1] Ferlay J (2015). Cancer incidence and mortality worldwide: sources, methods and major patterns in GLOBOCAN 2012. Int. J. Cancer.

[2] Bradley JD (2020). Long-term results of nrg oncology RTOG 0617: standard- versus high-dose chemoradiotherapy with or without cetuximab for unresectable stage iii non-small-cell lung cancer. J. Clin. Oncol.: Offic. J. Am. Soc. Clin. Oncol..

[3] Gray JE (2020). Three-year overall survival with durvalumab after chemoradiotherapy in stage III NSCLC-Update from PACIFIC. J. Thoracic Oncol.: Offic. Publ. Int. Assoc. Study Lung Cancer.

[4] Guberina M (2022). Effectiveness of durvalumab consolidation in stage III non-small-cell lung cancer: focus on treatment selection and prognostic factors. Immunotherapy.

[5] Girard N (2023). Treatment characteristics and real-world progression-free survival in patients with unresectable stage III NSCLC who received durvalumab after chemoradiotherapy: findings from the PACIFIC-R study. J. Thorac. Oncol. Offic. Publ. Int. Assoc. Study Lung Cancer.

[6] Chang AE, Piper-Vallillo AJ, Mak RH, Lanuti M, Muzikansky A, Rotow J, Jänne PA, Mino-Kenudson M, Swanson S, Wright CD, Kozono D (2024). The ASCENT Trial: a phase 2 study of induction and consolidation afatinib and chemoradiation with or without surgery in stage III EGFR-mutant NSCLC. Oncologist.

[7] Santoni M (2023). Complete remissions following immunotherapy or immuno-oncology combinations in cancer patients: the MOUSEION-03 meta-analysis. Cancer Immunol. Immunother.: CII.

[8] Rizzo A, Cusmai A, Giovannelli F, Acquafredda S, Rinaldi L, Misino A, Montagna ES, Ungaro V, Lorusso M, Palmiotti G (2022). Impact of proton pump inhibitors and histamine-2-receptor antagonists on non-small cell lung cancer immunotherapy: a systematic review and meta-analysis. Cancers.

[9] Rizzo A (2022). Identifying optimal first-line treatment for advanced non-small cell lung carcinoma with high PD-L1 expression: a matter of debate. British J. Cancer.

[10] Wang J (2021). Tislelizumab plus chemotherapy versus chemotherapy alone as first-line treatment for advanced squamous non-small-cell lung cancer: a phase 3 randomized clinical trial. JAMA Oncol..

[11] Wang Y (2022). Response to: Identifying optimal first-line treatment for advanced non-small cell lung carcinoma with high PD-L1 expression: a matter of debate. British J. Cancer.

[12] Wang Y (2022). Immune checkpoint inhibitors alone vs immune checkpoint inhibitors-combined chemotherapy for NSCLC patients with high PD-L1 expression: a network meta-analysis. British J. Cancer.

[13] Mandrekar SJ (2006). A prognostic model for advanced stage nonsmall cell lung cancer. Pooled analysis of North Central Cancer Treatment Group trials. Cancer.

[14] Tang C (2014). Lymphopenia association with gross tumor volume and lung V5 and its effects on non-small cell lung cancer patient outcomes. Int. J. Radiat. Oncol., Biol., Phys..

[15] Di Maio M (2005). Chemotherapy-induced neutropenia and treatment efficacy in advanced non-small-cell lung cancer: a pooled analysis of three randomised trials. Lancet. Oncol..

[16] Hoffmann M (2020). Blood parameters demonstrating a significant survival impact in patients with locally advanced NSCLC undergoing definitive chemoradiotherapy. Anticancer Res..

[17] Tolia M (2015). Prognostic significance of serum inflammatory response markers in newly diagnosed non-small cell lung cancer before chemoirradiation. BioMed Res. Int..

[18] Schussler O, Bobbio A, Dermine H, Lupo A, Damotte D, Lecarpentier Y, Alifano M (2022). Twenty-year survival of patients operated on for non-small-cell lung cancer: the impact of tumor stage and patient-related parameters. Cancers.

[19] Yang JR (2019). Post-diagnostic C-reactive protein and albumin predict survival in Chinese patients with non-small cell lung cancer: a prospective cohort study. Sci. Rep..

[20] Holgersson G (2017). The prognostic value of pre-treatment thrombocytosis in two cohorts of patients with non-small cell lung cancer treated with curatively intended chemoradiotherapy. Neoplasma.

[21] Huang Y (2022). The association between pretreatment anemia and overall survival in advanced non-small cell lung cancer: a retrospective cohort study using propensity score matching. J. Cancer.

[22] Guberina M, Pöttgen C, Guberina N, Hoffmann C, Wiesweg M, Richlitzki C, Metzenmacher M, Aigner C, Bölükbas S, Gauler T, Eberhardt WE (2024). Long-term survival in patients with oligometastatic non-small cell lung cancer by a multimodality treatment—comparison with stage III disease. Cancers.

[23] https://www.siemens-healthineers.com/de/integrated-chemistry/systems/atellica-solution-analyzers. (Access Date 13.02.2024).

[24] Guder WG (2002). Die Qualität diagnostischer Proben. Laboratoriums Medizin.

[25] Koul R, Rathod S, Dubey A, Bashir B, Chowdhury A (2018). Comparison of 7th and 8th editions of the UICC/AJCC TNM staging for non-small cell lung cancer in a non-metastatic North American cohort undergoing primary radiation treatment. Lung cancer (Amsterdam, Netherlands).

[26] Eberhardt WE (2015). Phase III study of surgery versus definitive concurrent chemoradiotherapy boost in patients with resectable stage IIIA(N2) and selected IIIB non-small-cell lung cancer after induction chemotherapy and concurrent chemoradiotherapy (ESPATUE). J. Clin. Oncol. Offic. J. Am. Soc. Clin. Oncol..

[27] Guberina M (2021). Prognostic value of post-induction chemotherapy volumetric pet/ct parameters for stage iiia/b non-small cell lung cancer patients receiving definitive chemoradiotherapy. J. Nucl. Med. Offic. Publ., Soc. Nucl. Med..

[28] Guberina M (2017). Heart dose exposure as prognostic marker after radiotherapy for resectable stage IIIA/B non-small-cell lung cancer: secondary analysis of a randomized trial. Ann Oncol. Offic. J. Eur. Soc. Med. Oncol..

[29] Simon RM, Subramanian J, Li MC, Menezes S (2011). Using cross-validation to evaluate predictive accuracy of survival risk classifiers based on high-dimensional data. Brief. Bioinform..

[30] Rushing C, Bulusu A, Hurwitz HI, Nixon AB, Pang H (2015). A leave-one-out cross-validation SAS macro for the identification of markers associated with survival. Comput. Biol. Med..

[31] Simonato L (2001). Lung cancer and cigarette smoking in Europe: an update of risk estimates and an assessment of inter-country heterogeneity. Int. J. Cancer.

[32] Antonia SJ (2017). Durvalumab after chemoradiotherapy in stage III non-small-cell lung cancer. N Engl. J. Med..

[33] CTCAE Version Classification v5.0, Common Terminology Criteria for Adverse Events (CTCAE) | Protocol Development | CTEP (cancer.gov): https://ctep.cancer.gov/protocolDevelopment/electronic_applications/ctc.htm#ctc_60, U.S. Department of Health and Human Services, National Institutes of Health, National Cancer Institute, USA.gov. (27.05.2024).

[34] van der Meer V, Neven AK, van den Broek PJ, Assendelft WJ (2005). Diagnostic value of C reactive protein in infections of the lower respiratory tract: systematic review. BMJ (Clinical research ed.).

[35] Scott HR (2002). The systemic inflammatory response, weight loss, performance status and survival in patients with inoperable non-small cell lung cancer. British J. Cancer.

[36] Murata D (2023). Survival and biomarkers for cachexia in non-small cell lung cancer receiving immune checkpoint inhibitors. Cancer Med..

[37] Zhang X, Huang JX, Tang M, Zhang Q, Deng L, Song CH, Li W, Shi HP, Cong MH (2024). Modified controlling nutritional status (mCONUT) serves as a promising prognostic factor in patients with cancer cachexia. Nutrition.

[38] Shukuya T (2023). Epidemiology, risk factors and impact of cachexia on patient outcome: results from the Japanese Lung Cancer Registry Study. J. Cachexia, Sarcopenia Muscle.

[39] Madeddu C, Busquets S, Donisi C, Lai E, Pretta A, López-Soriano FJ, Argilés JM, Scartozzi M, Macciò A (2023). Effect of cancer-related cachexia and associated changes in nutritional status, inflammatory status, and muscle mass on immunotherapy efficacy and survival in patients with advanced non-small cell lung cancer. Cancers.

[40] Kamp DW, Weitzman SA (2011). Chronic inflammation and cancer: the role of the mitochondria. Oncology.

[41] McMillan DC (2001). Measurement of the systemic inflammatory response predicts cancer-specific and non-cancer survival in patients with cancer. Nutr. Cancer.

[42] Xiao X, Wang S, Long G (2019). C-reactive protein is a significant predictor of improved survival in patients with advanced non-small cell lung cancer. Medicine.

[43] Kuusisalo S (2023). The prognostic and predictive roles of plasma C-reactive protein and PD-L1 in non-small cell lung cancer. Cancer Med..

[44] Hopkins AM (2020). Development and validation of a prognostic model for patients with advanced lung cancer treated with the immune checkpoint inhibitor atezolizumab. Clin. Cancer Res. Offic. J. Am. Assoc. Cancer Res..

[45] Minichsdorfer C, Gleiss A, Aretin MB, Schmidinger M, Fuereder T (2022). Serum parameters as prognostic biomarkers in a real world cancer patient population treated with anti PD-1/PD-L1 therapy. Ann. Med..

[46] Naqash AR, McCallen JD, Mi E, Iivanainen S, Marie MA, Gramenitskaya D, Clark J, Koivunen JP, Macherla S, Jonnalagadda S, Polsani S (2023). Increased interleukin-6/C-reactive protein levels are associated with the upregulation of the adenosine pathway and serve as potential markers of therapeutic resistance to immune checkpoint inhibitor-based therapies in non-small cell lung cancer. J. Immunother. Cancer.

[47] Guven DC (2022). The association between albumin levels and survival in patients treated with immune checkpoint inhibitors: A systematic review and meta-analysis. Front. Mol. Biosci..

[48] Zheng X (2023). Baseline C-reactive protein predicts efficacy of the first-line immune checkpoint inhibitors plus chemotherapy in advanced lung squamous cell carcinoma: a retrospective, multicenter study. BMC Cancer.

[49] Macciò A, Madeddu C (2020). Blocking inflammation to improve immunotherapy of advanced cancer. Immunology.

[50] Schuler M, Cuppens K, Plönes T, Wiesweg M, Du Pont B, Hegedus B, Köster J, Mairinger F, Darwiche K, Paschen A, Maes B (2024). Neoadjuvant nivolumab with or without relatlimab in resectable non-small-cell lung cancer: a randomized phase 2 trial. Nat. Med..

[51] Spils M (2024). Prognostic factors of recurrence and disease-free survival in radically resected pulmonary carcinoids: a real-world analysis. J. Thoracic Dis..

[52] Zaun G (2024). Comprehensive biomarker diagnostics of unfavorable cancer of unknown primary to identify patients eligible for precision medical therapies. Europe. J. Cancer.

[53] Di Federico A (2023). Predictors of survival to immunotherapy and chemoimmunotherapy in non-small cell lung cancer: a meta-analysis. J. Natl. Cancer Inst..

[54] Metzenmacher, M.*, et al.* Prognostic factors in nonsmall cell lung cancer: insights from the German CRISP registry. *The European respiratory journal* **61**(2023). 10.1183/13993003.01336-2022 PMC9892864 36180086

[55] Suazo-Zepeda E (2021). Risk factors for adverse events induced by immune checkpoint inhibitors in patients with non-small-cell lung cancer: a systematic review and meta-analysis. Cancer Immunol., Immunother. CII.

[56] Mitsuyoshi T (2018). Evaluation of a prognostic scoring system based on the systemic inflammatory and nutritional status of patients with locally advanced non-small-cell lung cancer treated with chemoradiotherapy. J. Radiat. Res..

[57] Guckenberger M (2020). Characterisation and classification of oligometastatic disease: a European society for radiotherapy and oncology and European organisation for research and treatment of cancer consensus recommendation. Lancet. Oncol..

[58] Stokke K (2022). Prognostic value of post first-line chemotherapy glasgow prognostic score in advanced non-small cell lung cancer. Clin. Med. Insights. Oncol..

[59] Sung M (2023). Prognostic value of baseline and early treatment response of neutrophil-lymphocyte ratio, C-reactive protein, and lactate dehydrogenase in non-small cell lung cancer patients undergoing immunotherapy. Translat. Lung Cancer Res..

[60] Saal J (2024). Integration of on-treatment modified Glasgow prognostic score (mGPS) to improve imaging-based prediction of outcomes in patients with non-small cell lung cancer on immune checkpoint inhibition. Lung Cancer (Amsterdam, Netherlands).

[61] Nassar YM, Ojara FW, Pérez-Pitarch A, Geiger K, Huisinga W, Hartung N, Michelet R, Holdenrieder S, Joerger M, Kloft C (2023). C-Reactive protein as an early predictor of efficacy in advanced non-small-cell lung cancer patients: a tumor dynamics-biomarker modeling framework. Cancers.

[62] Tanahashi M, Suzuki E, Yoshii N, Watanabe T, Tsuchida H, Yobita S, Iguchi K, Uchiyama S, Nakamura M (2022). Role of fluorodeoxyglucose-positron emission tomography in predicting the pathological response and prognosis after neoadjuvant chemoradiotherapy for locally advanced non-small-cell lung cancer. Interact. CardioVasc. Thoracic Surg..

[63] Aigner C, Hautzel H, Ploenes T (2022). SUVmax—Δ makes the difference. Interact. CardioVasc. Thoracic Surg..

[64] Zafra J, Onieva JL, Oliver J, Garrido-Barros M, González-Hernández A, Martínez-Gálvez B, Román A, Ordóñez-Marmolejo R, Pérez-Ruiz E, Benítez JC, Mesas A (2024). Novel Blood biomarkers for response prediction and monitoring of stereotactic ablative radiotherapy and immunotherapy in metastatic oligoprogressive lung cancer. Int. J. Mol. Sci..

[65] Rizzo A (2023). Hypertransaminasemia in cancer patients receiving immunotherapy and immune-based combinations: the MOUSEION-05 study. Cancer Immunol. Immunother. CII.

[66] Guberina M (2021). Impact of EBUS-TBNA in addition to [(18)F]FDG-PET/CT imaging on target volume definition for radiochemotherapy in stage III NSCLC. Europe. J. Nucl. Med. Mol. Imaging.

[67] Guberina M (2021). Patterns of nodal spread in stage III NSCLC: importance of EBUS-TBNA and (18)F-FDG PET/CT for radiotherapy target volume definition. Radiat. Oncol. (London, England).

[68] Pöttgen C (2016). Standardized uptake decrease on [18F]-fluorodeoxyglucose positron emission tomography after neoadjuvant chemotherapy is a prognostic classifier for long-term outcome after multimodality treatment: secondary analysis of a randomized trial for resectable stage IIIA/B non-small-cell lung cancer. J. Clin. Oncol. Offic. J. Am. Soc. Clin. Oncol..

[69] Guberina M (2022). Prediction of malignant lymph nodes in NSCLC by machine-learning classifiers using EBUS-TBNA and PET/CT. Sci. Rep..

