# Supplementary material for: C-reactive protein as robust laboratory value associated with prognosis in patients with stage III non-small cell lung cancer (NSCLC) treated with definitive radiochemotherapy
Source: Sci Rep. 2024 Jun 14;14:13765. doi: 10.1038/s41598-024-64302-2 (PMC11178931; doi:10.1038/s41598-024-64302-2)
Supplement: Supplementary file 1 — Supplementary Information. [file 41598_2024_64302_MOESM1_ESM.docx]

**Appendix**

| Laboratory parameters at initial diagnosis prior to induction chemotherapy (n=160) | | | | | | | |
| --- | --- | --- | --- | --- | --- | --- | --- |
|  |  | physiological value | CTCAE 1/2 | CTCAE 3 | CTCAE 4 | >ULN or <LLN | N/A |
|  | Median (Range) | Number % | Number % | Number % | Number % | Number % | Number % |
| Leukocytes | 9,00 (2,39-23,88) | 105 65,6 | 2 1,3 | 0 0,0 | 0 0,0 | 53 33,1 | 0 0,0 |
| Hb | 13,50 (7,8-17,9) | 88 55,0 | 71 44,4 | 1 0,6 | 0 0,0 | 0 0,0 | 0 0,0 |
| Thrombocytes | 312,50 (74-719) | 120 75,0 | 3 1,9 | 0 0,0 | 0 0,0 | 37 23,1 | 0 0,0 |
| Neutrophils | 5,90 (1,65-19,5) | 32 62,7 | 1 2,0 | 0 0,0 | 0 0,0 | 18 35,3 | 109 68,1 |
| Lymphocytes | 1,70 (0,05-5,9) | 36 70,6 | 8 15,7 | 0 0,0 | 1 2,0 | 6 11,8 | 109 68,1 |
| CRP | 1,50 (0,0-25,0) | 37 23,1 | 114 71,3 | 9 5,6 | 0 0,0 | 0 0,0 | 0 0,0 |
| LDH | 225 (114-1116) | 99 62,3 | 57 35,8 | 2 1,3 | 0 0,0 | 1 0,6 | 1 0,6 |
| ULN = upper limit of normal, LLN = lower limit of normal, >ULN or <LLN = if CTCAE not applicable, Hb = haemoglobin, N/A = not available, LDH = lactate dehydrogenase, CRP = C-reactive protein, CTCAE = Common Terminology Criteria for Adverse Events | | | | | | | |

Tabel 4: Laboratory parameters at initial diagnosis prior to induction chemotherapy

| Laboratory parameters prior to radiochemotherapy after induction chemotherapy (n=160) | | | | | | | |
| --- | --- | --- | --- | --- | --- | --- | --- |
|  |  | physiological value | CTCAE 1/2 | CTCAE 3 | CTCAE 4 | >ULN or <LLN | N/A |
|  | Median (Range) | Number % | Number % | Number % | Number % | Number % | Number % |
| Leukocytes | 7,00 (2,24-26,6) | 116 73,8 | 14 8,9 | 0 0,0 | 0 0,0 | 27 17,2 | 3 1,9 |
| Hb | 11,90 (6,2-16,6) | 30 19,1 | 125 79,6 | 1 0,6 | 0 0,0 | 1 0,6 | 3 1,9 |
| Thrombocytes | 259 (42-693) | 137 87,3 | 8 5,1 | 1 0,6 | 0 0,0 | 11 7,0 | 3 1,9 |
| Neutrophils | 4,29 (0,6-17,39) | 82 75,9 | 6 5,5 | 4 3,7 | 0 0,0 | 16 14,8 | 52 32,5 |
| Lymphocytes | 1,58 (0,56-4,78) | 81 75,0 | 17 15,7 | 0 0,0 | 0 0,0 | 10 9,3 | 52 32,5 |
| CRP | 0,65 (0,0-24,8) | 65 42,8 | 84 55,3 | 3 2,0 | 0 0,0 | 0 0,0 | 8 5,0 |
| LDH | 231 (153-1205) | 76 55,0 | 60 43,6 | 1 0,7 | 1 0,7 | 0 0,0 | 22 13,8 |
| ULN = upper limit of normal, LLN = lower limit of normal, >ULN or <LLN = if CTCAE not applicable, Hb = haemoglobin, N/A = not available, LDH = lactate dehydrogenase, CRP = C-reactive protein, CTCAE = Common Terminology Criteria for Adverse Events | | | | | | | |

Tabel 5: Laboratory parameters prior to radiochemotherapy after induction chemotherapy

| Laboratory parameters in the last week of radiotherapy (n=160) | | | | | | | |
| --- | --- | --- | --- | --- | --- | --- | --- |
|  |  | physiological value | CTCAE 1/2 | CTCAE 3 | CTCAE 4 | >ULN or <LLN | N/A |
|  | Median (Range) | Number % | Number % | Number % | Number % | Number % | Number % |
| Leukocytes | 4,43 (1,64-11,4) | 86 61,0 | 51 36,2 | 2 1,4 | 0 0,0 | 2 1,4 | 19 12,0 |
| Hb | 10,75 (6,4-16,6) | 14 10,1 | 121 87,0 | 3 2,2 | 0 0,0 | 1 2,2 | 21 13,1 |
| Thrombocytes | 210,50 (36-456) | 99 71,0 | 34 24,3 | 3 2,1 | 0 0,0 | 4 2,9 | 20 12,5 |
| Neutrophils | 3,01 (0,74-75,4) | 38 88,4 | 4 9,3 | 1 2,3 | 0 0,0 | 0 0,0 | 117 73,1 |
| Lymphocytes | 0,52 (0,16-3,18) | 3 7,0 | 19 44,2 | 17 39,5 | 3 7,0 | 1 2,3 | 117 73,1 |
| CRP | 1,20 (0,0-22,6) | 42 30,0 | 92 65,7 | 6 4,3 | 0 0,0 | 0 0,0 | 20 12,5 |
| LDH | 206 (13,6-502) | 46 66,7 | 23 33,3 | 0 0,0 | 0 0,0 | 0 0,0 | 91 57,0 |
| ULN = upper limit of normal, LLN = lower limit of normal, >ULN or <LLN = if CTCAE not applicable, Hb = haemoglobin, N/A = not available, LDH = lactate dehydrogenase, CRP = C-reactive protein, CTCAE = Common Terminology Criteria for Adverse Events | | | | | | | |

Tabel 6: Laboratory parameters in the last week of radiotherapy

| Laboratory parameters, nadir during radiochemotherapy (n=160) | | | | | | | |
| --- | --- | --- | --- | --- | --- | --- | --- |
|  |  | physiological value | CTCAE 1/2 | CTCAE 3 | CTCAE 4 | >ULN or <LLN | N/A |
|  | Median (Range) | Number % | Number % | Number % | Number % | Number % | Number % |
| Leukocytes | 2,83 (0,21-8,28) | 38 24,4 | 70 44,9 | 35 22,4 | 13 8,3 | 0 0,0 | 4 2,5 |
| Hb | 9,90 (5,0-17,1) | 5 3,2 | 124 79,5 | 27 17,3 | 0 0,0 | 0 0,0 | 4 2,5 |
| Thrombocytes | 143 (13-339) | 73 46,5 | 73 46,5 | 9 5,7 | 2 1,3 | 0 0,0 | 3 1,9 |
| Neutrophils | 2,35 (0,04-9,71) | 53 57,0 | 16 17,2 | 13 14,0 | 6 6,5 | 5 5,4 | 67 41,9 |
| Lymphocytes | 0,50 (0,06-9,0) | 12 13,0 | 33 35,5 | 35 37,6 | 9 9,7 | 2 2,2 | 67 41,9 |
| ULN = upper limit of normal, LLN = lower limit of normal, >ULN or <LLN = if CTCAE not applicable, Hb = haemoglobin, N/A = not available, LDH = lactate dehydrogenase, CRP = C-reactive protein, CTCAE = Common Terminology Criteria for Adverse Events | | | | | | | |

Tabel 7: Laboratory parameters, nadir during radiochemotherapy

| Laboratory parameters 6-8 weeks after radiochemotherapy (n=160) | | | | | | | |
| --- | --- | --- | --- | --- | --- | --- | --- |
|  |  | Normwertig | CTCAE 1/2 | CTCAE 3 | CTCAE 4 | >ULN or <LLN | N/A |
|  | Median (Range) | Anzahl % | Anzahl % | Anzahl % | Anzahl % | Anzahl % | Anzahl % |
| Leukocytes | 6,8 (3,31-16,39) | 65 89 | 2 2,7 | 0 0,0 | 0 0,0 | 15 20,5 | 78 49,0 |
| Hb | 12 (6,9-15,2) | 18 20,0 | 69 76,7 | 3 3,3 | 0 0,0 | 0 0,0 | 70 44,0 |
| Thrombocytes | 241,5 (57-529) | 80 90,0 | 3 3,4 | 0 0,0 | 0 0,0 | 6 6,7 | 71 44,4 |
| Neutrophils | 4,66 (2,05-14,7) | 49 83,0 | 0 0,0 | 0 0,0 | 0 0,0 | 10 17,0 | 101 63,1 |
| Lymphocytes | 0,82 (0,27-2,17) | 19 32,2 | 32 54,2 | 8 15,6 | 0 0,0 | 0 0,0 | 101 63,1 |
| CRP | 1,9 (0,0-22,3) | 25 28,1 | 55 61,8 | 9 10,1 | 0 0,0 | 0 0,0 | 71 44,4 |
| LDH | 244 (128-2130) | 39 49,4 | 38 48,1 | 0 0,0 | 2 2,5 | 0 0,0 | 81 51,0 |
| ULN = upper limit of normal, LLN = lower limit of normal, >ULN or <LLN = if CTCAE not applicable, Hb = haemoglobin, N/A = not available, LDH = lactate dehydrogenase, CRP = C-reactive protein, CTCAE = Common Terminology Criteria for Adverse Events | | | | | | | |

Tabel 8: Laboratory parameters 6-8 weeks after radiochemotherapy
